# The Mitogenome of *Sedum plumbizincicola* (Crassulaceae): Insights into RNA Editing, Lateral Gene Transfer, and Phylogenetic Implications

**DOI:** 10.3390/biology11111661

**Published:** 2022-11-13

**Authors:** Hengwu Ding, De Bi, Sijia Zhang, Shiyun Han, Yuanxin Ye, Ran Yi, Jianke Yang, Birong Liu, Longhua Wu, Renying Zhuo, Xianzhao Kan

**Affiliations:** 1 Anhui Provincial Key Laboratory of the Conservation and Exploitation of Biological Resources, College of Life Sciences, Anhui Normal University, Wuhu 241000, China; 2 College of Landscape Engineering, Suzhou Polytechnic Institute of Agriculture, Suzhou 215000, China; 3 CAS Key Laboratory of Soil Environment and Pollution Remediation, Institute of Soil Science, Chinese Academy of Sciences, Nanjing 210008, China; 4 State Key Laboratory of Tree Genetics and Breeding, Chinese Academy of Forestry, Hangzhou 311400, China; 5 Key Laboratory of Tree Breeding of Zhejiang Province, The Research Institute of Subtropical of Forestry, Chinese Academy of Forestry, Hangzhou 311400, China; 6 The Institute of Bioinformatics, College of Life Sciences, Anhui Normal University, Wuhu 241000, China

**Keywords:** *Sedum plumbizincicola*, Crassulaceae, mitogenome, RNA editing, gene transfer, phylogeny

## Abstract

**Simple Summary:**

Mitochondria are semiautonomous organelles in eukaryotic cells, which play a critical role in cellular energy production. The plant mitochondrial genomes harbor large degrees of variation and complexity in structures. Crassulaceae is the largest family in the order Saxifragales. However, no entire mitogenome data have been available for species of Crassulaceae up to now. In the present study, we sequenced the first mitogenome of Crassulaceae (presented by *Sedum plumbizincicola*). Through comprehensive analyses, we found Saxifragales mitogenomes have undergone rapid structural evolution, with low synonymous substitution rates. Moreover, RNA editing, gene transfer, secondary structures of mitochondrial RNAs, and phylogenetic implications were also analyzed by using mitochondrial data. The present work may provide new insights into the mitogenome evolution of Saxifragales.

**Abstract:**

As the largest family within the order Saxifragales, Crassulaceae contains about 34 genera with 1400 species. Mitochondria play a critical role in cellular energy production. Since the first land plant mitogenome was reported in *Arabidopsis*, more than 400 mitogenomic sequences have been deposited in a public database. However, no entire mitogenome data have been available for species of Crassulaceae to date. To better understand the evolutionary history of the organelles of Crassulaceae, we sequenced and performed comprehensive analyses on the mitogenome of *Sedum plumbizincicola*. The master mitogenomic circle is 212,159 bp in length, including 31 protein-coding genes (PCGs), 14 tRNA genes, and 3 rRNA genes. We further identified totally 508 RNA editing sites in PCGs, and demonstrated that the second codon positions of mitochondrial genes are most prone to RNA editing events. Notably, by neutrality plot analyses, we observed that the mitochondrial RNA editing events have large effects on the driving forces of plant evolution. Additionally, 4 MTPTs and 686 NUMTs were detected in the mitochondrial and nuclear genomes of *S. plumbizincicola*, respectively. Additionally, we conducted further analyses on gene transfer, secondary structures of mitochondrial RNAs, and phylogenetic implications. Therefore, the findings presented here will be helpful for future investigations on plant mitogenomes.

## 1. Introduction

Mitochondria are semiautonomous organelles in eukaryotic cells, which play a critical role in cellular energy production. In contrast to the small compacted circular mitogenomes in animals [1,2,3], plant mitogenomes exhibit different evolutionary patterns, with high rearrangement, low mutation rate, and large size [4,5,6]. For currently known seed plants, the smallest mitogenome was detected in a parasitic species *Viscum scurruloideum* Barlow, with a length of 66 kb and many genes missing [7], while the largest was from *Larix sibirica* Ledeb. (11.7 Mb) [8]. Generally, plant mitogenomes contain some dispersed repeat sequences (several kb in size), which have both forward and reverse orientations [9]. Moreover, these repeats frequently recombine, resulting in isomerized mitogenomes [5,10,11]. Typically, angiosperm mitogenomes hold 24 core genes (mostly coding for respiratory proteins) and 17 non-core genes (also called variable genes, mostly encoding ribosomal proteins), together with 3–30 tRNA and three rRNA genes [12,13,14,15].

RNA editing is a post-transcriptional process, where some nucleotides in the mature RNAs differ from their genomic templates by nucleotide indels or conversions [16,17]. Mitochondrial PCGs generally need to be RNA edited to perform their functions [18]. Owing to the universal cytidine (C) to uridine (U) conversions by deamination in plants, RNA editing could alter the encoded amino acids [19], and influence the codon bias of PCGs to some extent [20].

During evolutionary history, lateral gene transfer events usually happened from the organelle genomes to the nuclear genome, as well as between the plastome and the mitogenome [21,22,23]. Plastid-derived insertions in mitogenomes are known as mitochondrial plastid sequences (MTPTs) [24]. With a few exceptions of tRNA genes, most MTPTs lose their functions and are considered as pseudogenes [24]. Meanwhile, mitochondrial-derived insertions in nuclear genomes are called nuclear mitochondrial sequences (NUMTs) [25]. In most cases, NUMTs have been reported to be nonfunctional in the nucleus of plants [26,27,28]. Occasionally, some functionally transferred genes that are usually lost in mitogenomes are detected for several mitochondrial ribosomal protein genes (*rps10*, *rps14*, and *rps19*) and succinate dehydrogenase genes in angiosperms [22,29,30,31].

Crassulaceae is the largest family in the order Saxifragales, containing about 34 genera with close to 1400 species [32,33]. Since the first land plant mitogenome was reported in *Arabidopsis* L. [10], more than 400 mitogenomic sequences have been deposited at NCBI (accessed October 2022). However, no entire mitogenome data have been available for species of Crassulaceae up to now. *Sedum plumbizincicola* X.H.Guo & S.B.Zhou ex L.H.Wu, a perennial Crassulaceae species, is newly reported from lead–zinc mining areas in Zhejiang province, China [34]. Although *S. plumbizincicola* is notable for its Zn/Cd hyperaccumulation ability [34,35,36,37,38], its exact taxonomic status remains unclear. Knowledge of plant mitogenomes might provide new insights into the evolutionary history of *S. plumbizincicola*.

Recently, high-throughput sequencing technologies provided unique opportunities to obtain the mitogenomes of plants. The combined utilization of long reads (Oxford Nanopore Technology, ONT) and short reads (Illumina technology) can greatly improve the continuity and completeness of mitogenome assembly [39]. In the current study, we sequenced the mitogenome of *S. plumbizincicola* by using these two technologies. Together with the public data, we performed comparative analyses of mitogenomes within Saxifragales. Consequently, the aims of this study were: (1) features of *S. plumbizincicola* mitogenome, (2) the influences of RNA editing events, (3) characteristics of gene transfer events, (4) tRNA and rRNA secondary structures, and (5) mitophylogenetic relationships within the order Saxifragales.

## 2. Materials and Methods

### 2.1. Sampling, DNA Extraction, and Sequencing

The sample of *S. plumbizincicola* (Code: AHNU-KPBK001) was obtained from Panjiacun in Zhejiang Province of China (latitude 29°35′16″ N, longitude 118°35′19″ E). In order to predict high-quality mitogenomic sequences, and to identify possible NUMTs, we employed short-read and long-read sequencing technologies (Illumina and Nanopore, respectively). Total genomic DNA extraction was conducted with a Plant Genomic DNA kit (Tiangen Biotech, Beijing, China). The libraries were prepared by using TruSeq DNA PCR-Free Library Prep Kit (Illumina, San Diego, CA, USA) for short reads and ONT Ligation Sequencing Kit 1D (Nanopore, Oxford, UK) for long reads. Subsequently, two libraries were sequenced on the Illumina Hiseq X Ten (Illumina, San Diego, CA, USA) and Oxford Nanopore GridION X5 (Nanopore, Oxford, UK), respectively.

### 2.2. Mitogenome Assembly, and Gene Annotation

Two strategies were applied to obtain the reliable *S. plumbizincicola* mitogenome. In the first strategy, the ONT reads were corrected and de novo assembled by using Canu v1.7.1 [40] and SMARTdenovo 1.0 [41], respectively. The assembled genome was polished three times by Pilon v.1.22 [42] with Illumina short reads. Then, we used BLASTn 2.9.0 [43] to identify the mitogenome from the polished genome. In the second strategy, Illumina short reads were assembled using GetOrganelle toolkit 1.7.1 [44] with the mitogenome obtained from the previous step as a reference template. By comparing the assemblies from the two strategies, we finally obtain a master circle of the *S. plumbizincicola* mitogenome.

The sequencing depth of the mitogenome was measured with bowtie 2.4.1 [45] and minimap 2.17 [46]. Then, the complete mitogenome was annotated with GeSeq [47]. Dispersed and tandem repeats were detected with ROUSFinder.py [48] and Tandem Repeats Finder 4.09 [49], respectively. Furthermore, the collinearity analyses of *S. plumbizincicola mitogenome* with its close species (*Heuchera parviflora* var. *saurensis* R.A.Folk, KR559021) were carried out using the progressive mauve algorithm implemented in MAUVE v.2.4.0 with default parameters [50].

To better measure the overall nucleotide substitution rates of mitochondrial genes within *S. plumbizincicola* and *H. parviflora*, plastid genes were selected as references. Thus, two datasets were prepared. The first dataset consists of 29 concatenated mitochondrial PCGs. The other includes 79 combined plastid PCGs (data were retrieved from our previous research [51]). The nonsynonymous substitutions rates (dN), synonymous substitutions rates (dS), and their ratios (ω) were calculated with PAML v4.9 (The ω > 1, =1, and <1 indicate positive, neutral, and purifying selection, respectively) [52].

### 2.3. RNA Editing Sites Identification and Codon Usage Analysis

Eight transcriptomic data of *S. plumbizincicola* (Accession Number: SRR5118121-SRR5118128) were retrieved from THE NCBI SRA database and assembled by Trinity v2.8.5 [53] for RNA editing sites identification.

In the present study, the codon usage analyses contained the following factors: the effective number of codons (ENC), GC content at codon sites 1 and 2 (GC12), 3 (GC3), and synonymous 3 (GC3s), ENC-GC3s plot, and neutrality plot. Except for three stop codons (TAA, TAG, and TGA), the ENC and GC3s were performed in DnaSP 6.12.03 [54]. The GC12 and GC3 were calculated by using MEGA X 10.0.5 [55]. In addition, the ENC-GC3s plot analysis has proven to be a highly efficient tool for verifying the main driving factor of mutation pressure or natural selection [56,57,58,59,60]. Meanwhile, the neutrality plot (GC12 vs. GC3) can be used to estimate the extent of directional mutation pressure against natural selection in the codons. In this plot, the regression coefficient (absolute slop) is regarded as the mutation-selection equilibrium coefficient (1 for the complete mutational bias and 0 for complete natural selective constraint) [61,62]. Here, all plots were drawn with R x64 4.0.2. Due to the ubiquitous RNA editing phenomena, we also conducted comparative analyses of all PCGs before and after RNA editing.

### 2.4. Identification of Gene Transfer

MTPTs and NUMTs were identified by using BLASTn with our previous plastomic data (MN185459) [51] as a reference and our current nuclear genome (unpublished), respectively. The lost mitochondrial non-core genes were detected in nuclear genome and transcriptomes by using BLASTn with corresponding reference sequences from mitogenomes of *H. parviflora* (KR559021) [63], *Vitis vinifera* L. (NC_012119) [64], and *Amborella trichopoda* Baill. (KF754803) [65].

### 2.5. Structure Prediction of tRNAs and rRNAs

Cloverleaf structures of tRNAs were reconstructed by using tRNAscan-SE 2.0 [66]. For 5S rRNA, secondary structures were predicted by comparison with the generalized 5S rRNA model proposed by Delihas and Andersen [67]. In addition, the structural templates of 18S and 26S rRNA were obtained from the Comparative RNA Web Site (CRW) [68].

### 2.6. Mitophylogenetic Analysis

Because of the limited availability of mitochondrial sequences within Saxifragales, we additionally retrieved data from publically assembled mitochondrial scaffolds and the SRA database (Table S1). Mitophylogenetic relationships among Saxifragales species were reconstructed using the maximum-likelihood (ML) and Bayesian inference (BI) methods based on conserved regions (totally 34 coding sequences and 16 introns). Recently, phylogenomic study has unraveled that Saxifragales is sister to Rosids [69]. Therefore, two Rosids species (*Gossypium barbadense* L., NC_028254; *G. hirsutum* Cav., NC_027406) were selected as outgroups. Multiple sequence alignment was carried out using MUSCLE v3.8 [70]. The ML analyses were performed using RAxML 8.2.12 [71]. Node support was evaluated by 1000 thorough bootstrap replicates (under the GTRCAT model) with 100 random starting trees. Moreover, the convergence of ML analyses was carried out using the RAxML package with the parameter “-I autoMRE”. For BI analysis, the best-fit model for each locus was selected based on Bayesian information criterion (BIC) values by using ModelTest-NG 0.1.6 [72]. Subsequently, the BI analyses with two simultaneous runs and four independent Markov chains (10,000,000 generations, sampling every 1000th generations) were performed by using MrBayes 3.2.7a [73]. The convergence of the BI analyses was checked with Tracer 1.7.1 [74].

## 3. Results

### 3.1. Genome Features of Mitogenome

**Figure 1:**
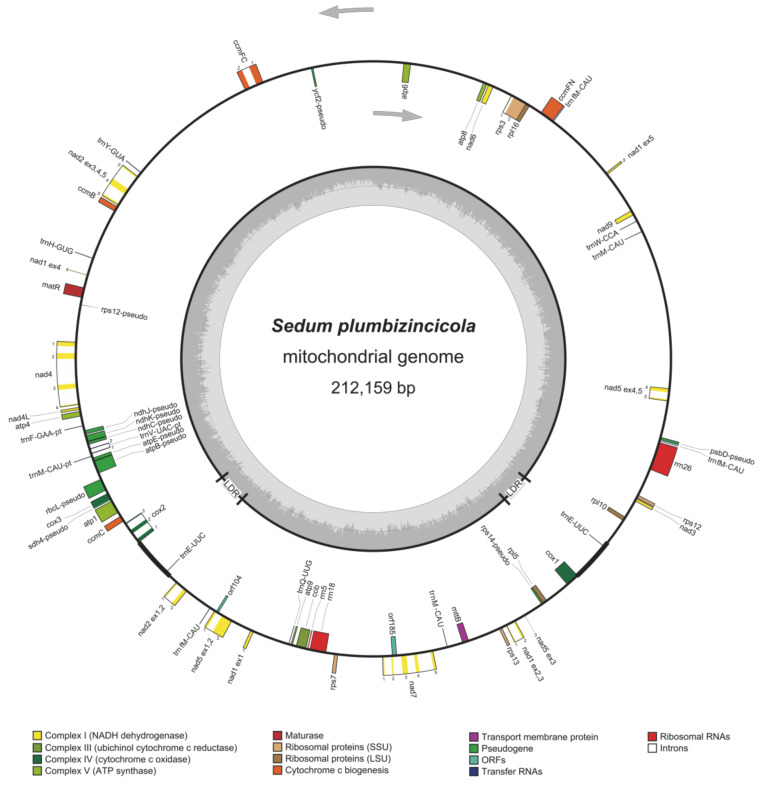
Mitogenome annotation map for *S. plumbizincicola*. Genes lying outside the circle are transcribed in a clockwise direction, whereas genes inside are transcribed in a counterclockwise direction. The dashed darker and lighter gray in the inner circle denote G + C and A + T contents of mitochondrial genome, respectively. LDRs mean large direct repeats.

Totally, 32 Gb of Illumina sequences (91,076,287 paired-end clean reads) and 7 Gb of corrected ONT data (137,808 corrected long reads) were generated, respectively. The assembly results from two methods were deposited in Data S1. According to our assembly strategies, a master circle (MC) of *S. plumbizincicola* mitogenome (Figure 1) was obtained, with 212,159 bp in size and 44.5% GC content (Accession Number: OP588116). In total, 1,820,050 Illumina paired reads (2.00% of total reads, 2512.43× mean coverage) and 22,844 corrected ONT reads (16.58% of total reads, 280.53× mean coverage) were mapped to the master circle of mitogenome using bowtie2 and minimap2 tools (Figure S1 and Table S2).

The mitogenome of *S. plumbizincicola* consists of 24 core genes and 7 non-core genes. The proteins encoded by these core genes were identified, including nine complex I subunits (ND1–7, 9, and 4L), one complex III subunit (COB), three complex IV subunits (CO1–3), five complex V subunits (ATP1, 4, 6, 8 and 9), and four subunits involved in the biogenesis of cytochrome c (ccmB, ccmC, ccmFN, and ccmFC), maturase (matR), and transport membrane protein (mttB). In contrast, all non-core genes (*rps3*, *rps7*, *rpl10*, *rps12*, *rps13*, *rpl5*, and *rpl16*) encode ribosomal proteins. Additionally, the mitogenome contains 14 tRNA genes (11 for native mitochondrial tRNAs and 3 for plastome-derived tRNAs), 3 rRNA genes (*rrn5*, *rrn18*, and *rrn26*), along with 2 intronic ORFs and 11 clear pseudogenes (9 plastome-derived pseudogenes and 2 native mitochondrial pseudogenes). Moreover, we also detected two identical large direct repeats (LDRs, 5222 bp), three small tandem repeats (<200 bp), and abundant small dispersed repeats (<200 bp) in this mitogenome (Table S3).

Based on recombination via LDRs, the mitochondrial MC of *S. plumbizincicola* could generate two subgenomic circles: SC1 (156,162 bp) and SC2 (55,997 bp) (Figure S2 and Data S2). In order to check the existence of three conformations (MC, SC1, and SC2), we further checked sequences of the LDRs and their flanking regions (FRs) (extracted 1 kb nucleotides for each FR) in ONT long reads. If three conformations existed in vivo together, four types of boundary sequences should be detected among ONT reads: type 1 (FR1-LDR-FR2), type 2 (FR3-LDR-FR4), type 3 (FR1-LDR-FR4), and type 4 (FR3-LDR-FR2). The type 1 and type 2 boundary sequences belonged to MC. The type 3 and type 4 boundary sequences existed in SC1 and SC2, respectively. Fortunately, the ONT reads containing four types of boundary sequences were detected together (Figure S2 and Data S2), which might indicate the coexistence of three mitochondrial conformations.

**Figure 2:**
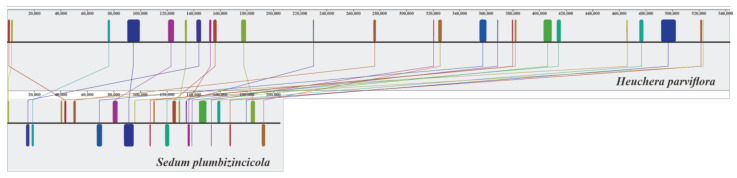
Mitogenome synteny between *S. plumbizincicola* and *H. parviflora*. The sequence of *H. parviflora* was selected as reference. The minimum locally collinear block size was 1006. The homologous regions shown with same colors.

The general features of *S. plumbizincicola* mitogenome compared to those of its close species are summarized in Table 1. The size of *S. plumbizincicola* is nearly 2.6-fold shorter than that of *H. parviflora* (Saxifragales). The functional gene number of *S. plumbizincicola* is less than that of *H. parviflora*. Nevertheless, *S. plumbizincicola* has more ORFs and pseudogenes within the mitogenome than *H. parviflora*. Notably, the size and proportion of repeats within *S. plumbizincicola* mitogenome are relatively lower by contrast with *H. parviflora*. In addition, compared with animal mitogenomes, all investigated plant mitogenomes had high-proportioned noncoding regions. According to the syntenic plot (Figure 2), no obvious synteny was observed between mitogenomes of *S. plumbizincicola* and *H. parviflora*.

Moreover, we compared the dN, dS, and ω values between mitochondrial and plastid genes within *S. plumbizincicola* and *H. parviflora* at overall levels. It is clear that these two organelle PCGs (both ω < 1) have been under purifying selection, since they diverged from their closest common ancestor (Table 2). Notably, the dN and dS values of concatenated mitochondrial genes are 1.9-fold and 4.6-fold lower than those of concatenated plastid genes (Table 2), respectively.

### 3.2. RNA Editing Sites and Codon Usage Pattern

**Figure 3:**
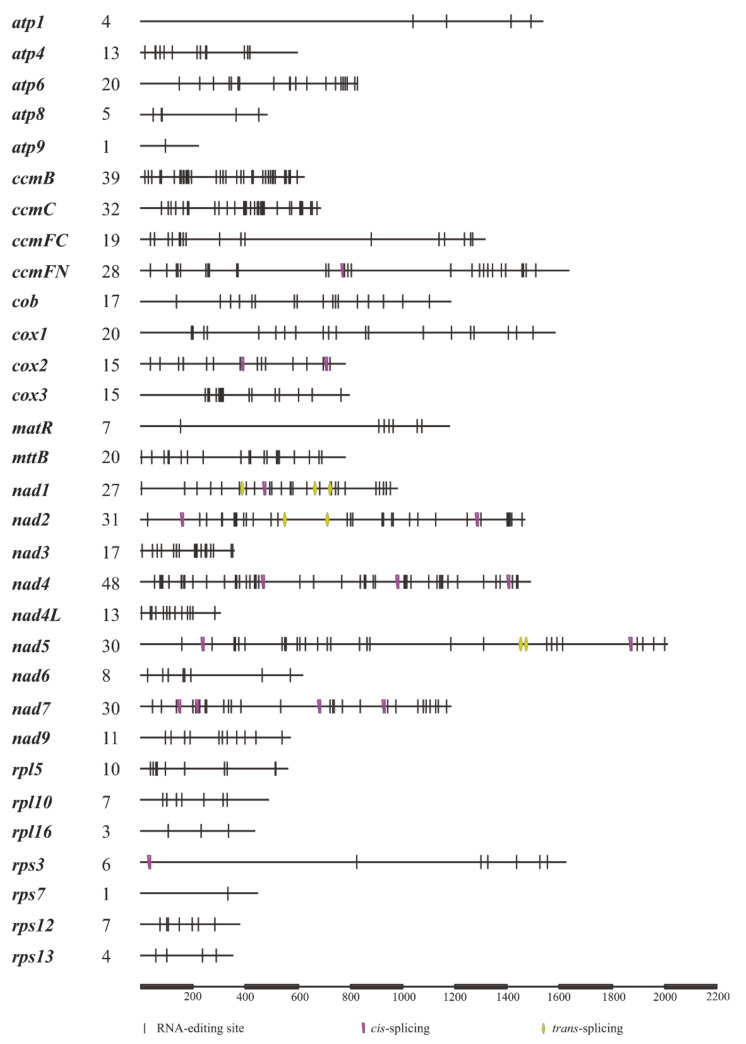
Localization of RNA editing sites in mitochondrial PCGs within *S. plumbizincicola*.

After aligning the genomic sequences with their corresponding cDNA regions (Data S3), totally, 508 RNA editing sites were identified in the 31 PCGs (Table S4). Among these sites, 30.12%, 63.78%, and 6.10% occurred in the 1st, 2nd, and 3rd positions of codons, respectively. As illustrated in Figure 3, *nad4* had the highest density of RNA editing sites (48 sites), followed by *ccmB* (39 sites) and *ccmC* (32 sites). In addition, it is worth noting that no RNA editing events were found in tRNA and rRNA genes.

Further analysis showed that these editing sites totally changed 496 codons, including 474 nonsynonymous and 22 synonymous codons (Table S4). These edited codons could result in 20 types of amino acid conversion. Among them, the top three conversion events were Ser → Leu (22.98%), Pro → Leu (22.58%), and Ser → Phe (14.72%) (Table 3). The codon counts and codon ratios of PCGs before and after RNA editing were summarized in Table 4. Due to the high rates of Ser → Leu and Pro → Leu, as well as the low rate of Leu → Phe (3.02%), 211 codons encoding for Leu increased after RNA editing totally (Table 4). As expected, the usages of codons encoding for Pro and Ser were accordingly decreased (Table 4). Notably, we also observed that RNA editing created initiation and termination codons for PCGs. For instance, initiation codons for *nad1* and *nad4* (ACG → AUG) and termination codons for *atp6* (CAA → UAA) were generated by RNA editing.

**Figure 4:**
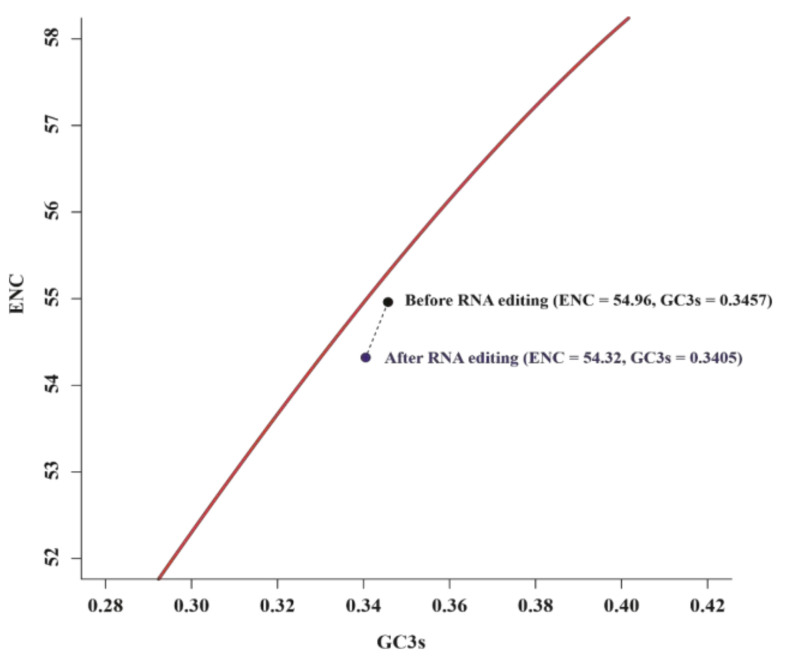
The ENC-GC3s plot of mitochondrial PCGs at overall level before and after RNA editing. The red line represents the expected ENC curve (ENC_expected_ = 2 + GC3s + 29/[GC3s^2^ + (1 − GC3s)^2^]).

Due to the codon conversion, RNA editing could change the codon usage indexes, such as ENC, GC3s, GC12, and GC3 values of PCGs. These results were listed in Table S5. Generally, ENC values ≤ 35 indicate high codon preference [56,59,60]. In the present analyses, the ENC values of all 31 PCGs both before (39.09–61) and after RNA editing (38.15–61) were higher than 35, which indicates mitochondrial genes of *S. plumbizincicola* lacked strong codon bias. In the ENC-GC3s plot (Figure 4), two points fell below the expected curve, indicating that the overall PCGs were mainly under the influence of natural selection before and after RNA editing. Moreover, our results indicated that RNA editing events could slightly reduce the overall ENC (54.96 → 54.32) and GC3s (0.3457 → 0.3405) values.

**Figure 5:**
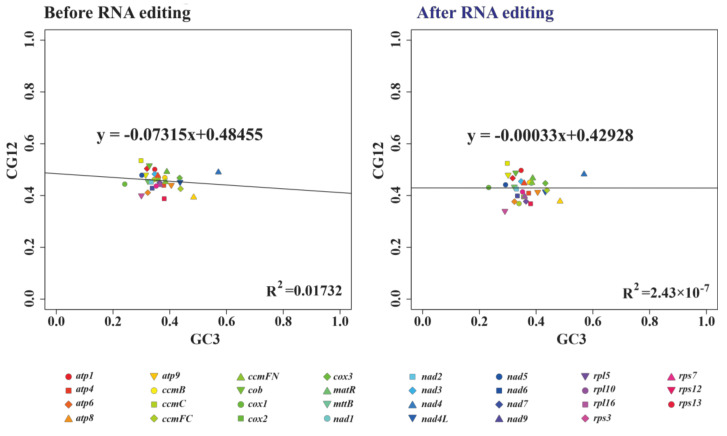
Neutrality plots of mitochondrial PCGs before and after RNA editing.

In addition, the regression coefficient (0.07315) before RNA editing in the neutrality plot showed that the contribution of mutation pressure was 7.315% (Figure 5). By contrast, that value had decreased dramatically (0.00033) after RNA editing, implying the degree of mutation pressure dropped to 0.033% (Figure 5).

### 3.3. Identification of Gene Transfer

To investigate lateral gene transfer events in *S. plumbizincicola* mitogenome, we further analyzed in detail the features of MTPTs and NUMTs. At first, four MTPTs were detected in the *S. plumbizincicola* mitogenome, with lengths ranging from 394 to 8411 bp (Table 5). The identities between these MTPTs and their corresponding plastid sequences ranged from 86.38% to 93.72% (Table 5). Notably, the longest MTPT in *S. plumbizincicola* shared an identical gene order with its corresponding plastid sequence, harboring six complete PCGs and three tRNA genes. In order to further confirm this transfer event, we analyzed the FRs of ONT reads across this transferred sequence. The results conducted by BLASTn showed that all FRs hit the mitogenome, while no FR hit the plastome (Table S6), manifesting that the mitochondrial assembly is accurate. Thus, this long MTPT is indeed present in the mitogenome of *S. plumbizincicola*. Owing to frameshift mutations and internal termination codons, all the six PCGs might be pseudogenized (Figure S3). In contrast, no variation was detected in the above three tRNA genes. The remaining three MTPTs containing incomplete plastid genes (*rps12*, *psbD*, and *ycf2*) were also likely to be nonfunctional (Table 5).

Moreover, a total of 686 NUMTs were identified in our assembled nuclear genome. The sizes and sequence identities of NUMTs were in the range of 33–15,124 bp and 69.65–100%, respectively (Table S7). Most of them (684) were derived from the noncoding region or partial genes of the mitogenome, with only two exceptions (NUMT395 and NUMT656), which contained complete *matR* and *ccmFC*, respectively. Compared with mitochondrial homologous genes, NUMT395 and NUMT656 also might be pseudogenized for many indels and mutations (Figure S4).

Furthermore, many observations in plants have substantiated that some non-core genes lost in the mitogenome could transfer to the nuclear genome [75,76,77,78]. From further comparative analyses, we found that 10 non-core genes (*rps1*, *rps2*, *rps4*, *rps10*, *rps11, rps14*, *rps19*, *rpl2*, *sdh3*, *sdh4*) might have been lost in the mitogenome of *S. plumbizincicola*. Notably, with the exception of *rps4*, *rps11*, and *sdh4* genes, the remaining 7 non-core genes were detected in both genomes (Accession Number: OP558021–OP558029) and transcriptome data (Figure S5). Moreover, the *rps14* and *rps19* genes from the nuclear genome have two heterogeneous copies. The mean sequencing depths of these transferred non-core genes ranged from 120.9–246.44× (estimated by Illumina clean reads) to 22.23–79.5× (estimated by corrected ONT reads), respectively (Table S2). The mean sequencing depths of these transferred genes were much lower than those of mitochondrial MC, indicating the assemblies of mitogenome and transferred non-core genes are reliable.

### 3.4. Secondary Structures of Mitochondrial RNAs

**Figure 6:**
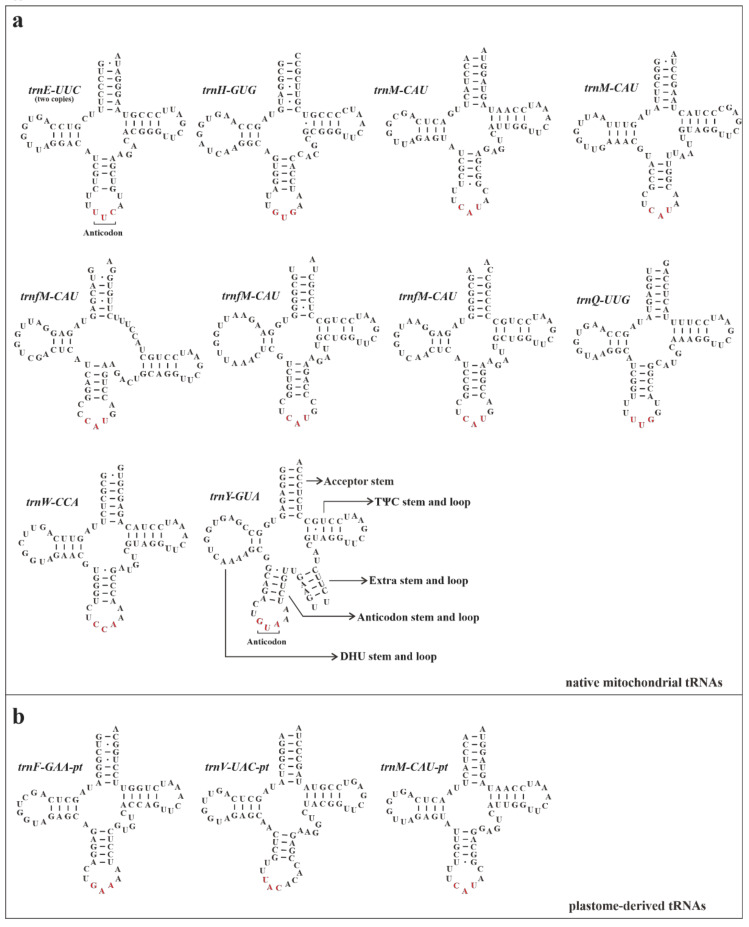
The predicted secondary structures of tRNAs found in mitogenome of *S. plumbizincicola*: (**a**) native mitochondrial tRNAs; (**b**) plastome-derived tRNAs.

All native mitochondrial tRNAs (mt-tRNAs) could be folded into canonical cloverleaf secondary structures (Figure 6a). The *trnE-UUC*, which is located in the LDR regions, had two identical native copies. The *trnM-CAU* and *trnfM-CAU*, respectively, had two and three different native copies in the mitogenome. Three plastome-derived tRNA genes (*trnF-GAA-pt*, *trnM-CAU-pt*, and *trnV-UAC-pt*) seemingly had a normal function, because they had the potential to form the right secondary structures (Figure 6b).

**Figure 7:**
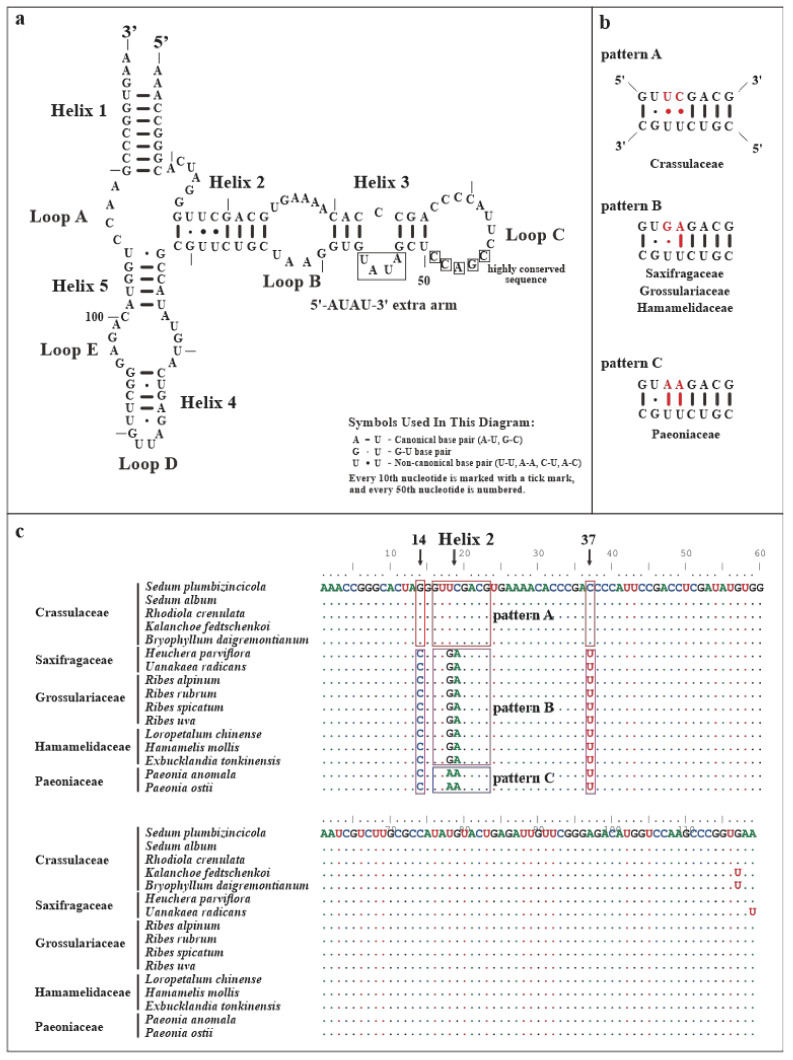
The predicted secondary structures and sequences of 5S mtrRNAs. (**a**) The secondary structures of 5S mtrRNAs of *S. plumbizincicola.* (**b**) The different structural patterns of Helix 2 within Saxifragales. (**c**) The sequences of 5S mtrRNAs investigated within Saxifragales.

**Figure 8:**
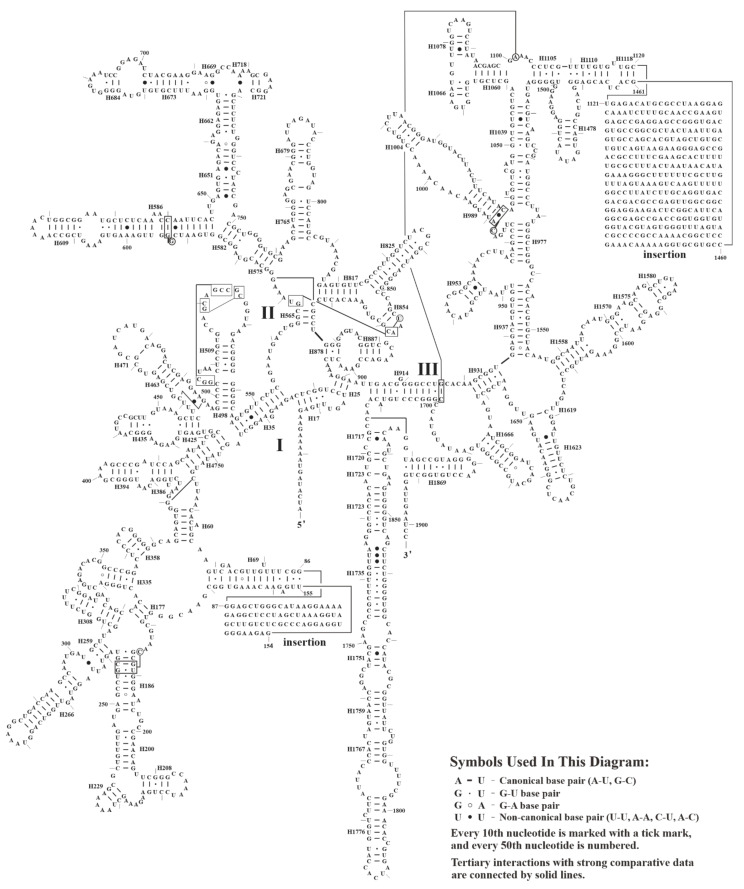
The predicted secondary structure of 18S mtrRNA of *S. plumbizincicola*.

Additionally, the secondary structure of *S. plumbizincicola* mtrRNAs were established. The sizes of 5S, 18S, and 26S mtrRNAs are 119, 1902, and 3261 nucleotides (nts) respectively. The secondary structure of 5S mtrRNA consists of five helices and five loops (one hinge region, two hairpins, and two internal loops) (Figure 7a). In particular, one 5′-AUAU-3′ extra arm was found adjacent to 5’-CGACC-3’ block, which is highly conserved and can interact with aminoacyl-tRNA (Figure 7a) [79]. Unlike base pairs (G-U and A-U) observed in *Oenothera* L., *Triticum aestivum* L., and *Silene latifolia* Poir. [79,80,81], two mismatched pairs (U-U and C-U) were detected in Helix 2 from *S. plumbizincicola* (Figure 7a). To avoid a potential sequencing error, we confirmed these two mismatched pairs by more comparative analyses of transcriptomic data from *S. plumbizincicola* (Accession Number: SRR5118121–SRR5118128). Then, 5S mtrRNAs sequences of 16 different species representing 5 families of Saxifragales were retrieved and analyzed (Data S4). Most notably, three pairing patterns were detected in the Helix 2 of 5S mtrRNAs within Saxifragales: Pattern A (U-U and C-U), Pattern B (G-U and A-U), and Pattern C (double A-U) (Figure 7b,c). Five Crassulaceae species and two Paeoniaceae species belonged to Patterns A and C, respectively. In addition, nine species from the other three families were categorized into Pattern B. Moreover, two uniform substitutions of 5S mtrRNAs were also observed in Crassulaceae (Position 14: C → G, and Position 37: U → C) (Figure 7c). Moreover, the 18S and 26S mtrRNA of *S. plumbizincicola* have 3 domains (75 helices) (Figure 8) and 6 domains (130 helices) (Figure S6), respectively. Notably, four insertions were detected in the mtrRNAs: two in 18S mtrRNA (68 nts for Domain I and 340 nts for Domain III) and two in 26S mtrRNA (386 nts for Domain I and 530 nts for Domain III). According to a proposal by Chao et al. [82], these insertions are not secondary structures in rRNA.

Further, we compared the sequence identities of mtrRNAs between *S. plumbizincicola* and *H. parviflora*. Results from Table 6 indicated that the identities of 5S, 18S, and 26S mtrRNA were 96.64%, 97.16%, and 88.16%, respectively. Clearly, the 26S mtrRNA was the most divergent among the three types of mtrRNAs. Within the domains of 18S and 26S mtrRNAs, Domains II and VI had the highest identities, respectively. Interestingly, for insertions, the identities of 26S mtrRNA were much lower than those of 18S mtrRNA.

### 3.5. Mitophylogenetic Implications

**Figure 9:**
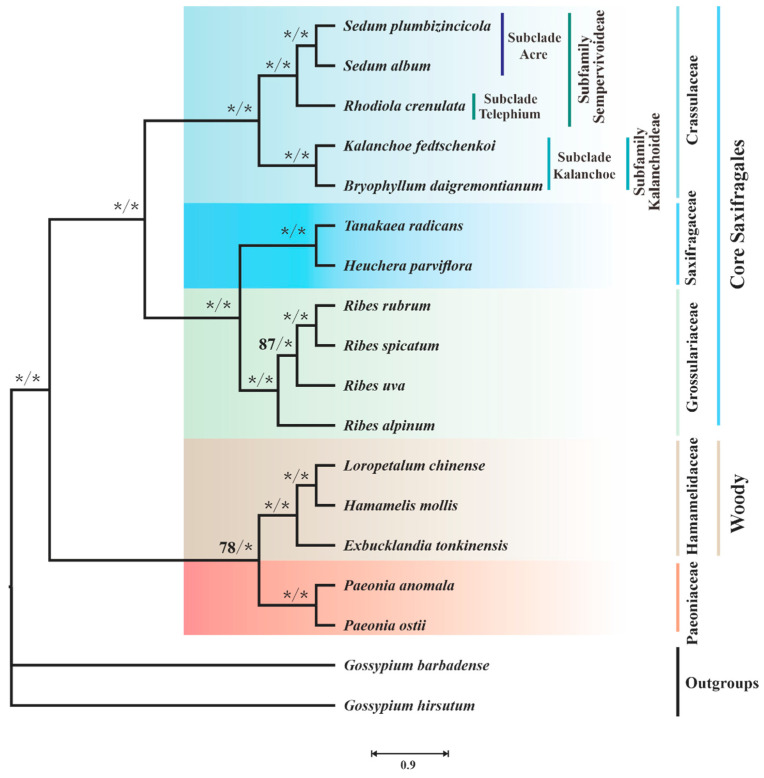
Mitophylogenetic tree of Saxifragales species. This analysis is based on 34 PCGs (CDS) and 16 introns. The maximum-likelihood bootstrap (BS) and bayesian posterior probability (PP) values for each node are indicated. * indicates 100% BS or 1.00 PP.

To investigate the mitophylogeny of Saxifragales, 16 species representing 5 families, plus 2 Rosids species as outgroups, were employed in the analyses (Data S5). The best-fit model of each partition can be seen in Table S8. The concatenated sequence matrix was 60,184 bp long. ML and BI analyses yielded nearly identical trees. The effective sample size (ESS) measured by Tracer was equal to 7550 (ESS >> 200), indicating the BI analyses were convergent. As shown in Figure 9, species of Saxifragales, with a limited sample size, could be clustered into two clades: core Saxifragales clade and Paeoniaceae plus the woody clade.

Within the former clade, Crassulaceae is monophyletic and sister to the Saxifragaceae alliance (Saxifragaceae and Grossulariaceae) with high supports (maximum-likelihood bootstrap (BS) = 100 and bayesian posterior probability (PP) = 1.0). Two subfamilies Sempervivoideae and Kalanchoideae belong to the Crassulaceae family. Within the Sempervivoideae, two species of the genus *Sedum* (*S. plumbizincicola* and *S. album* L.) are sister to *Rhodiola crenulate* H.Ohba. Additionally, *Kalanchoe fedtschenkoi* Raym.-Hamet & H.Perrier, and *Bryophyllum daigremontianum* Raym.-Hamet & H.Perrier formed a distinct subclade (Kalanchoe) (BS= 100 and PP= 1.00). Moreover, Saxifragaceae, represented by *H. parviflora* and *Tanakaea radicans* Franch. & Sav., have a sister relationship with Grossulariaceae, including four *Ribes* species. In the clade of Paeoniaceae plus the woody, the sister relationship between Paeoniaceae and Hamamelidaceae was strongly supported by BI analysis and only weakly supported by the ML method (BS = 78 and PP = 1.00).

## 4. Discussion

In our present study, we reported the first mitogenome of Crassulaceae. Comprehensive analyses were carried out on the mitogenome of *S. plumbizincicola*, including basic genomic characteristics, RNA editing sites, gene transfer events, secondary structures of RNAs, and mitophylogeny. The present work reports new insights into the mitogenome evolution of Saxifragales.

As previous studies reported, angiosperm mitogenomes had extensive structural variations, such as high rearrangement rates, and enormous diversity in genomes sizes [12,81,83,84,85]. For instance, within the genus *Silene*, the mitogenomes may vary over 40-fold in size and display almost no conserved synteny [12,81,83,84]. Nevertheless, the mitochondrial genes have extremely low synonymous substitution rates in angiosperms [86,87,88,89]. As reported by Drouin et al. [88], the dS value (0.128 ± 0.005) of mitochondrial genes (3 genes) is approximately 3-fold lower than that (0.388 ± 0.012) of plastid genes (5 genes) in 17 species of angiosperms. Here, we observed a high degree of structural differences between the mitogenomes of *S. plumbizincicola* and *H. parviflora*. For these two species, the very low dS value (0.0697) of concatenated mitochondrial genes (29 genes) was found, which is 4.6-fold lower than that (0.319) of plastid genes (79 genes). Moreover, these peculiar characteristics obtained by this work might be explained by abundant double-strand break repair (DSBR) in plant mitochondria [48,90,91]. DSBR is very accurate when the repair is template-based, resulting in the low substitution rate in genes. On the other hand, DSBR, which rely on the nonhomologous end-joining (NHEJ) or break-induced replication (BIR) pathways, can account for the size expansions and loss of synteny through rearrangements [48,90,91]. Therefore, based on more loci, our findings here strongly suggest that Saxifragales mitogenomes have also undergone rapid structural evolution, as well as low synonymous substitution rates.

Large repeats in plant mitogenomes play a crucial part in inter- or intramolecular recombination [9,92,93,94]. Recombination between large inverted repeats (LIRs) and large direct repeats (LDRs) can redistribute sequences (‘flip-flop’) and generate circular isoforms (‘loop-outs’), respectively [93,94,95,96]. In this study, a pair of LDRs were detected in the mitochondrial MC of *S. plumbizincicola*, which might generate two additional isoforms (SC1 and SC2). Moreover, ONT sequencing supported the presence of recombination mediated by LDRs, implying the existence of these two putative isoforms. Similar phenomena that multi-isoforms were generated by mitochondrial LDRs have been reported in many plants, such as in *Arabidopsis* [93], *Oryza sativa* L. [97], and *Zea mays* L. [96]. Hence, our results manifested LDRs could affect the structural dynamics of *S. plumbizincicola* mitogenome.

RNA editing plays a pivotal role in the regulation of mitochondrial gene expression [98,99,100]. In plant organelles, most RNA editing causes C-to-U substitutions (higher plants) [17,21,101,102,103,104,105,106,107,108,109] and occasionally U-to-C conversions (basal plants) [110,111]. Editing sites exhibited a highly uneven distribution (with frequencies at the codon positions: 2nd > 1st >> 3th), which is highly conserved among angiosperms [17,21,105,109]. Our present study first examined the RNA editing sites in mitochondrial genes of *S. plumbizincicola*: a total of 508 C-to-U editing sites (no U-to-C) were identified, and most of them presented at the 2nd and 1st positions of codons. These findings further demonstrated that the second codon positions of mitochondrial genes are most prone to RNA editing events [17,21,105,109].

Most noticeably, this uneven distribution results in many nonsynonymous amino acid conversions, such as Ser → Leu, Pro → Leu, and Ser → Phe in the mitochondrial PCGs of *S. plumbizincicola*. These three conversion types are also conserved among angiosperms [21,109]. It is interesting why RNA editing mainly caused nonsynonymous amino acid substitutions. A hypothesis put forward by Gualberto et al. [112] considered that RNA editing is a universal correction mechanism. RNA editing effectively suppresses the effect of DNA mutations, because most editing events can restore amino acids that are conserved in nonediting plants and in their bacterial ancestors [99,112,113]. In particular, the neutrality plot analysis could be used to quantify the extent of mutation against natural selection [61,62]. However, this analysis approach was only performed in a few plant mitogenomes at the genomic level [56,114]. Taking *S. plumbizincicola* as an example, our study executed neutrality plot analyses at both genomic and transcriptomic levels. Surprisingly, it is clear that the effect degree of mutation pressure in the mitogenome of *S. plumbizincicola* dropped from 7.315% (before RNA editing) to 0.033% (after RNA editing). Our results illustrated that the mitochondrial RNA editing events have large effects on the driving forces of plant evolution.

Further, the gene loss events usually happened in angiosperm mitogenomes [21,115,116,117]. In our study, totally, 10 non-core genes were lost within the *S. plumbizincicola* mitogenome. To explain this mitochondrial gene loss, three fundamental reasons were presented: (1) the lost genes are unnecessary in the mitogenome, (2) the functions of lost genes are replaced by other genes, and (3) the lost genes are transferred into the nucleus [76,115]. Here, we found seven mitochondrial non-core genes have migrated into the nuclear genome of *S. plumbizincicola*. This finding implied that lateral gene transfer might have occurred in most lost genes from plant mitogenomes.

According to the Angiosperm Phylogeny Group (APG) system IV [118], the order Saxifragales includes 15 families. Our mitophylogenetic tree divided 16 species into two clades and five families, which were generally congruent with the framework phylogeny of Saxifragales reported by Folk et al. (nuclear data) [119], Ding et al. (plastid data) [51], and Han et al. (plastid data) [120]. Nevertheless, there are still some unsolved phylogenetic problems within Saxifragales. For example, the exact taxonomic position of *S. plumbizincicola* is not entirely clear. Ding et al. [51] indicated that the *S. plumbizincicola* mitogenome was close to *S. sarmentosum* Bunge. Han et al. [120] subsequently implied that *S. plumbizincicola* had a closer relationship with *S. tricarpum* Makino than *S. sarmentosum* by increasing sampling size. Different from those two results, our current study displays that the closest species of *S. plumbizincicola* is *S. album*. These inconsistent results were mainly caused by limited data. In addition, the deep relationships inferred by mitochondrial data within Crassulaceae or Saxifragales are largely unknown. In order to clear the exact taxonomic status of *S. plumbizincicola*, and understand the phylogeny of Crassulaceae or Saxifragales, more data are needed for further comprehensive analyses.

## 5. Conclusions

This study presented the first mitogenome of Crassulaceae. The mitogenome of *S. plumbizincicola*, with 212,159 bp in size and 44.5% GC content, includes 31 PCGs, 14 tRNAs, 3 rRNA, 2 ORFs, and 11 pseudogenes. The PCGs contain 508 RNA editing sites, changing 496 codons. Most of the changing codons belong to nonsynonymous conversions. RNA editing dramatically decreased the effect of DNA mutations. Next, 4 MTPTs and 686 NUMTs were detected in the mitogenome and the nuclear genome, respectively. Moreover, our study indicated that seven lost mitochondrial non-core genes have transferred to the nuclear genome. By contrast, we found the synonymous substitution rate of mitochondrial genes was 4.6-fold lower than that of plastid genes at overall levels between *S. plumbizincicola* and its close species. In addition, we focused on the analyses of the secondary structures of mitochondrial RNAs. Notably, we found the Helix 2 regions of 5S mtrRNAs are more divergent among Saxifragales. Based on the 34 coding sequences and 16 introns from 16 species, phylogenetic analyses displayed that *S. plumbizincicola* had a closer relationship with *S. album* than other Crassulaceae species. Our findings will be useful for further analyses of the evolution of mitogenome, including RNA editing, gene transfer, RNA secondary structure, and phylogeny.

## Figures and Tables

**Table 1:** Comparisons of general features of mitogenomes between *S. plumbizincicola* and *H. parviflora*.

Species	*Sedum plumbizincicola*	*Heuchera parviflora*
Accession	OP588116	KR559021
Size (bp)	212,159	542,954
GC content	44.51%	45.75%
Functional genes	48	77
tRNAs	14	28
rRNAs	3	7
PCGs	31	42
ORFs	2	0
Pseudogenes	11	7
Coding region (bp)	33,814 (15.93%)	45,189 (8.32%)
Noncoding region (bp)	178,345 (84.07%)	497,765 (91.68%)
Dispersed repeats (bp)	12,884 (6.07%)	119,727 (22.05%)
Tandem repeats (bp)	129 (0.06%)	1459 (0.27%)
Plastid-derived sequences (bp)	10,394 (4.90%)	25,562 (4.71%)

**Table 2:** Substitution rates of overall mitochondrial genes and plastid genes between *S. plumbizincicola* and *H. parviflora*.

Gene	ω	dN	dS
Concatenated mitochondrial genes	0.4188	0.0292	0.0697
Concatenated plastid genes	0.1763	0.0562	0.3190

**Table 3:** Amino acid conversion types caused by RNA editing.

AA Conversion	Count (Ratio)	Conversion Type
Ser → Leu	114 (22.98%)	Nonsynonymous
Pro → Leu	112 (22.58%)	Nonsynonymous
Ser → Phe	73 (14.72%)	Nonsynonymous
Pro → Ser	42 (8.47%)	Nonsynonymous
Arg → Cys	37 (7.46%)	Nonsynonymous
Arg → Trp	32 (6.45%)	Nonsynonymous
His → Tyr	21 (4.23%)	Nonsynonymous
Leu → Phe	15 (3.02%)	Nonsynonymous
Thr → Ile	9 (1.81%)	Nonsynonymous
Ala → Val	6 (1.21%)	Nonsynonymous
Thr → Met	6 (1.21%)	Nonsynonymous
Pro → Phe	6 (1.21%)	Nonsynonymous
Gln → Termination	1 (0.20%)	Nonsynonymous
Leu → Leu	6 (1.21%)	Synonymous
Phe → Phe	5 (1.01%)	Synonymous
Ile → Ile	3 (0.60%)	Synonymous
Tyr → Tyr	3 (0.60%)	Synonymous
Pro → Pro	2 (0.40%)	Synonymous
Val → Val	2 (0.40%)	Synonymous
Ser → Ser	1 (0.20%)	Synonymous

**Table 4:** Comparisons of codon usage before and after RNA editing.

Amino Acid	Codon	Genomic DNA		Change after Editing	
		Count	Ratio	Count	Ratio
Ala	GCA	151	1.65%	−1	−0.01%
	GCC	126	1.38%	−1	−0.01%
	GCG	74	0.81%	−3	−0.03%
	GCU	248	2.71%	−1	−0.01%
Arg	AGA	115	1.26%	0	0
	AGG	66	0.72%	0	0
	CGU	128	1.40%	−28	−0.31%
	CGC	58	0.63%	−9	−0.10%
	CGA	119	1.30%	0	0
	CGG	85	0.93%	−32	−0.35%
Asn	AAC	81	0.88%	0	0
	AAU	203	2.22%	0	0
Asp	GAC	90	0.98%	0	0
	GAU	202	2.20%	0	0
Cys	UGC	56	0.61%	+9	+0.10%
	UGU	79	0.86%	+28	+0.31%
Gln	CAA	206	2.25%	−1	−0.01%
	CAG	57	0.62%	0	0
Glu	GAA	253	2.76%	0	0
	GAG	111	1.21%	0	0
Gly	GGA	240	2.62%	0	0
	GGC	89	0.97%	0	0
	GGG	123	1.34%	0	0
	GGU	212	2.31%	0	0
His	CAC	42	0.46%	−6	−0.07%
	CAU	175	1.91%	−15	−0.16%
Ile	AUA	202	2.20%	+4	+0.04%
	AUC	194	2.12%	-2	-0.02%
	AUU	333	3.63%	+7	+0.08%
Leu	CUA	131	1.43%	+34	+0.37%
	CUC	98	1.07%	+4	+0.04%
	CUG	88	0.96%	+32	+0.35%
	CUU	205	2.24%	+20	+0.22%
	UUA	250	2.73%	+75	+0.82%
	UUG	183	2.00%	+46	+0.50%
Lys	AAA	233	2.54%	0	0
	AAG	125	1.36%	0	0
Met	AUG	244	2.66%	+6	0.07%
Phe	UUC	247	2.70%	+25	0.27%
	UUU	348	3.80%	+69	0.75%
Pro	CCA	144	1.57%	−45	−0.49%
	CCC	118	1.29%	−26	−0.28%
	CCG	87	0.95%	−41	−0.45%
	CCU	184	2.01%	−48	−0.52%
Ser	AGC	89	0.97%	0	0
	AGU	149	1.63%	0	0
	UCA	175	1.91%	−64	−0.70%
	UCC	133	1.45%	−15	−0.16%
	UCG	110	1.20%	−38	−0.41%
	UCU	189	2.06%	−28	−0.31%
Thr	ACA	117	1.28%	−4	−0.04%
	ACC	123	1.34%	−1	−0.01%
	ACG	72	0.79%	−6	−0.07%
	ACU	157	1.71%	−4	−0.04%
Trp	UGG	141	1.54%	+32	0.35%
Tyr	UAC	69	0.75%	+3	0.03%
	UAU	226	2.47%	+18	0.20%
Val	GUA	172	1.88%	+1	0.01%
	GUC	103	1.12%	−1	−0.01%
	GUG	127	1.39%	+3	0.03%
	GUU	178	1.94%	+3	0.03%
Termination	UAA	19	0.21%	+1	0.01%
	UAG	6	0.07%	0	0
	UGA	5	0.05%	0	0

**Table 5:** Identified MTPTs in *S. plumbizincicola* mitogenome.

MTPT Regions	Mitogenome Coordinates	MTPT Size (bp)	MTPT GC Content (%)	Plastome Coordinates	Plastomic Sequence Size (bp)	Plastomic Sequence GC Content (%)	Identity (%)	MTPT Annotations
MTPT1	43,609–44,639	1031	36.86	88,171–89,250 (−)	1080	37.31	86.38	*ycf2*-partial
MTPT2	83,645–84,202	558	38.71	66,564–67,129 (−)	566	38.52	89.02	*rps12*-partial
MTPT3	96,864–105,274	8411	37.00	45,747–54,551 (+)	8805	36.72	93.72	*trnF-GAA, ndhJ, ndhK, ndhC, trnV-UAC, trnM-CAU, atpE, atpB, rbcL*
MTPT4	186,562–186,955	394	44.42	31,555–31,958 (+)	404	42.08	90.59	*psbD*-partial

**Table 6:** Identities of mtrRNA between *S. plumbizincicola* and *H. parviflora*.

Domain	Identity (%)		
	5S mtrRNA	18S mtrRNA	26S mtrRNA
overall	96.64	97.16	88.16
domain I (insertion)		98.94 (97.09)	79.91 (40.56)
domain II		99.14	98.70
domain III (insertion)		95.50 (88.24)	76.13 (65.66)
domain IV			98.64
domain V			98.96
domain VI			99.59

## Data Availability

The sequence data generated in this study are available in GenBank of the National Center for Biotechnology Information (NCBI) under the access numbers: OP558021–OP558029, and OP588116.

## Supplementary Materials

The following supporting information can be downloaded at: https://www.mdpi.com/article/10.3390/biology11111661/s1, Figure S1: Depths of the assembled mitogenome using Illumina reads and ONT reads; Figure S2: Mitochondrial conformations of *Sedum plumbizincicola*; Figure S3: The comparisons of MTPT sequences and their corresponding plastid genes; Figure S4: The comparisons of NUMT sequences and their corresponding mitochondrial genes; Figure S5: The homologous nuclear copies of mitochondrial non-core genes that lost in mitogenome of *Sedum plumbizincicola*; Figure S6: The predicted secondary structure of 26S mtrRNA of *Sedum plumbizincicola*; Table S1: The accession numbers of species selected in the mitophylogenetic analyses; Table S2: Sequencing depths of mitogenome and transferred non-core genes; Table S3: Identified repeats in *Sedum plumbizincicola* mitogenome; Table S4: The information of RNA editing sites; Table S5: The ENC, GC3s, GC12, and GC3 values of PCGs before and after RNA editing; Table S6: The hit results of flanking regions of ONT reads across the transferred sequence; Table S7: Determination of NUMTs in *Sedum plumbizincicola* nuclear genome; Table S8: The best Bayesian evolutionary models of each dataset; Data S1: Comparisons of assembly results of two strategies; Data S2: The sequences of master circle and two subgenomic circles; Data S3: The sequences of mitochondrial PCGs before and after RNA editing; Data S4: 5S mtrRNA sequences of 16 investigated species; Data S5: Mitophylogenetic datasets of 34 CDS and 16 introns.
