# Supplementary material for: The Mitogenome of Sedum plumbizincicola (Crassulaceae): Insights into RNA Editing, Lateral Gene Transfer, and Phylogenetic Implications
Source: Biology (Basel). 2022 Nov 13;11(11):1661. doi: 10.3390/biology11111661 (PMC9687357; doi:10.3390/biology11111661)
Supplement: Supplementary file 1 [file biology-11-01661-s001.zip › Figure S1.pdf]

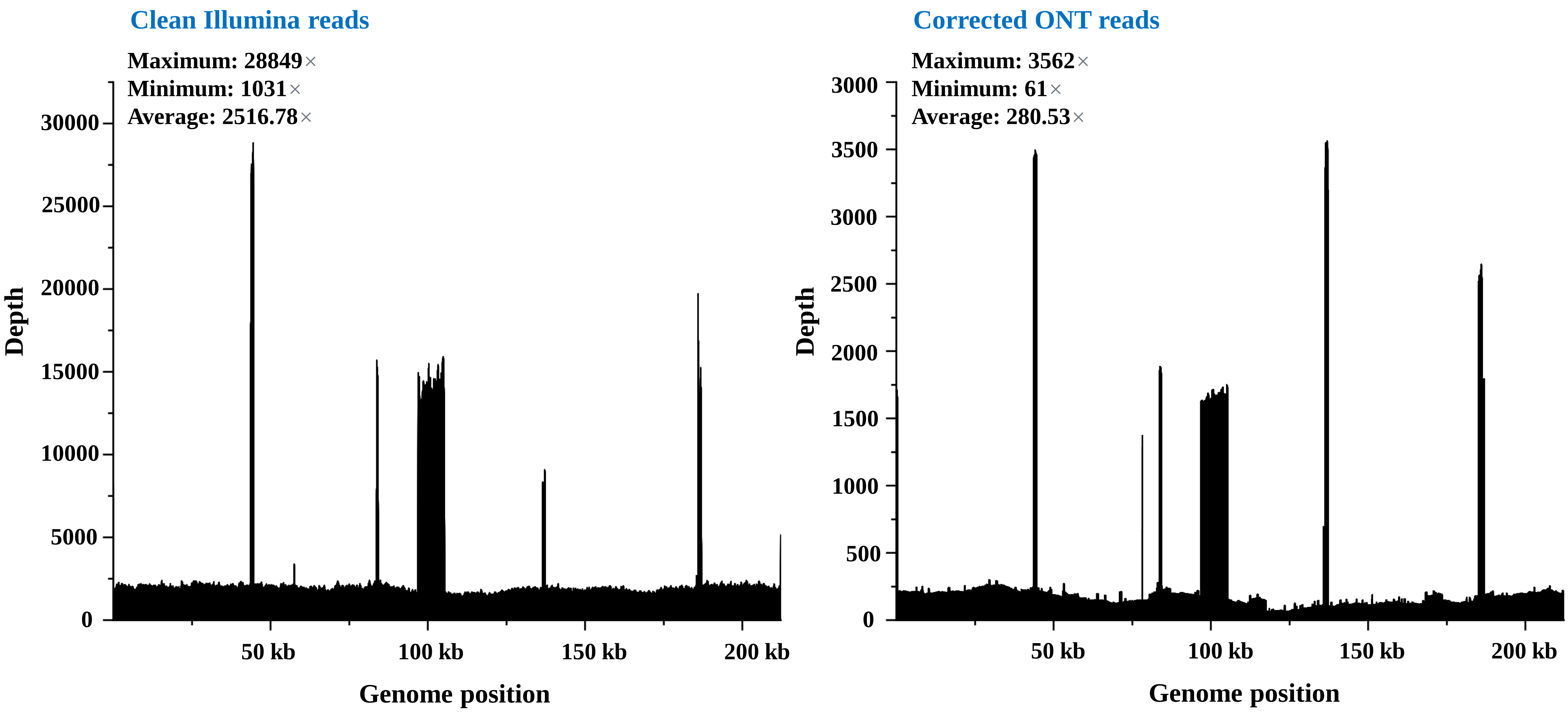
Clean Illumina reads
Maximum: 28849×
Minimum: 1031×
Average: 2516.78×
30000
25000
20000
15000
10000
5000
0
Depth
50 kb
100 kb
150 kb
200 kb
Genome position
Corrected ONT reads
Maximum: 3562×
Minimum: 61×
Average: 280.53×
3000
3500
3000
2500
2000
1500
1000
500
0
Depth
50 kb
100 kb
150 kb
200 kb
Genome position
