# Supplementary material for: The Mitogenome of Sedum plumbizincicola (Crassulaceae): Insights into RNA Editing, Lateral Gene Transfer, and Phylogenetic Implications
Source: Biology (Basel). 2022 Nov 13;11(11):1661. doi: 10.3390/biology11111661 (PMC9687357; doi:10.3390/biology11111661)
Supplement: Supplementary file 1 [file biology-11-01661-s001.zip › Figure S3.pdf]

```
                  10        20        30        40        50        60        70        80        90       100
atpB         MRISPTTSDPGISAFKKKHLGRIVQIIGPVLDVVFSPGNMPNIYNALVVKGRDITGQQINVTCEVQQLLGNNRVRAVAMSATDGLMRGMEVVDTGAPLSV
atpB-pseudo  MRISPTTSDPGISAFKKKHLGRIVQIIGPVLDVVFSPGNMPNIYNALVVKGRDITGQQINVTCEVQQLLGNNRVRAVAMSATDGLMRGMEVVDTGAPLSV

                 110       120       130       140       150       160       170       180       190       200
atpB         PVGGVTLGRIFNVLGEPVDNLGPVDTGTTSPIHRSAPAFIQLDTKLSIFETGIKVVDLLAPYRRGGKIGLFGGAGVGKTVLIMELINNIAKAHGGVSVFG
atpB-pseudo  PVGGVTLGRICNVLGEPVDNLGPVDTGTTSPIHRSAPAFIQLDTKFSILKQ*SS--RSFSPLWRKNRT--IRGAGVGKTVLIMELINNIAKAHGGVSVFG

                 210       220       230       240       250       260       270       280       290       300
atpB         GVGERTREGNDLYMEMKESGVINEKNIAESKVALVYGQMNEPPGARMRVGLTALTMAEYFRDVNEQDVLLFIDNIFRFVQAGSEVSALLGRMPSAVGYQP
atpB-pseudo  GVGERTREGNDLYMEMKESGVINEKNIAE*KVALVYGQMNEPPGARMRVGLTALTMAEYFRDVNEQDVLFFIDNIFRFVQAGSEVSALLGRMPSAVG*KT

                 310       320       330       340       350       360       370       380       390       400
atpB         TLSTEMGSL-------QERITSTKEGSITSIQAVYVP--ADDLTDPAPATTFAHLDATTVLSRGLAAKGIY-------PAVDPLDSTSTMLQPRIVGEEH
atpB-pseudo  YP*Y*NGFFTRKNYFYQRRVYNFYSSCLCTCRRFD*PCACNDIC--AFRCYYCTIKRISC-*RYLSSSGSFRFNVNYAPTSDCW*GT---L*NCSKS*GN

                 410       420       430       440       450       460       470       480       490       500
atpB         YETAQRVKETLQRYKELQDIIAILGLD-ELSEEDRL----TVARARKIERFFSQPFFVA----EVFTGSPGKYVGLVETIRGFKLILSGELDSLPEQAFY
atpB-pseudo  FTTLQRTSRHYS-YPWVGRIIRRGSFNGSTSNQNETFFLPTLFRSRSFYRFSRKICWFSRNN*RV*INSLG---GII*S----------------S*AGF

                 510       520
atpB         LVGNIDEATAKAVNLEMESKSNK*---
atpB-pseudo  LFGR*HRRSYRE-GCELRNGEQIE*MX
```

```
                  10        20        30        40        50        60        70        80        90       100
atpE         MTFNLCVLTPNRIVWDSEVKEIILSTNSGQIGVLPNHAPIATAVDIGILRIRLNNE---------WLTMALM-------GGFARIGNNTITILVNDAEKG
atpE-pseudo  MTFNLCVLTLIELFGIPK*KK------SSYLLIVDKLAYYQTTLLLLQL*I*GF*EYALTTNG*RWL*WAVLLE*VIMRSLF**MMQRRVVTLI------

                 110       120       130       140       150       160
atpE         SDIDPQEAQQTLEKAETNLQKAEGKRQTIEANLALRRARTRVEAINTIS*----------------
atpE-pseudo  ----PKKPNKLLK-----------KRKRI-----CRKRKVRDKQLRQI*LSDELGHE*RLSIQFQX
```

```
                  10        20        30        40        50        60        70        80        90       100
ndhC         MFLLYEYDIFWAFLIISSVIPIVVFLISGVLATISKGPEKLSSYESGIEPMGDAWLQFRIRYYMFALV-FVVFDVETVFLYPWAMSFDVVGVSVFIEALI
ndhC-pseudo  MFLLYEYDIFWAFLIISSVIPIVAFRISGVLATISKGLEKLSSYESGIEPMGDAWLQFRIRYYIICLL*FLLFLMLKLFFFIPGQ*VLM*WVYPYL*KLS

                 110       120
ndhC         FVLI--LIVGSVYAWRKGALEWS*--
ndhC-pseudo  FSCLS*LLVQFMHGEKE---HWNGLX
```

```
                  10        20        30        40        50        60        70        80        90       100
ndhJ         MQGRLSAWLVKHGLVHRSLGFDYQGIETLQIKSEDWHSIAVILYVYGYNYLRSQCAYDVAPGGMLASVYHLTRIEYGVDQPEEVCIKVFVPRKSPRIPSV
ndhJ-pseudo  MQGRLSAWLVKHGLVHRSLGFDYQGIETLQIKSEDWHSIAVILYVYGYNYLRSQCAYDVAPGGMLASVYHLTRI*YGVDQPEEVCIKVFVPRKSPRIPSV

                 110       120       130       140       150
ndhJ         FWVWKSSDFQERESYDMLGIYYDNHPRLKRILMPESWIGWPLRKDYIAPNFYEIQDAH*
ndhJ-pseudo  FWVWKSADFQERESYDMLGIYYDNHPRLKRILMPESWIGWPLRKDYIAPNFYEIQDAH*
```

```
                  10        20        30        40        50        60        70        80        90       100
ndhK         MVLAPEFSDNKKNKVKKKKLETVMNSIEFPLVNRTTQNS--------------VIST-----TS-------------NDLSNWSRLSSLWPLLYGTSCCF
ndhK-pseudo  MVLAPEFADNKKNKVKKK----A*DSYEFH*VSFS*SNNPKFSYFNYIK*SFQLVKTLQFMATSLRYQLLLH*ICFINRLT--IRL*SFWT---GTK---

                 110       120       130       140       150       160       170       180       190       200
ndhK         IEFASLIGSRFDFDRFGLVPRSSPRQADLILTAGTVTMKMAPSLVRLYEQMPEPKYVIAMGACTITGGMFSTDSYSTVRGVDKLIPVDVYLPGCPPKPEA
ndhK-pseudo  IES*T---SRPNFN-------SGYR----------------------YDE---------------NGSLFS-----------------------------E

                 210       220       230       240       250       260       270       280       290       300
ndhK         IIDALTKLRKKISR---EIYDDRIRSQ-----QGNRCFTTNHK--FEVGRSTHTG------------NYDQGLLYQSP---------YTSEISLETFFKY
ndhK-pseudo  II*A--NARTKICYCYGGLYNYRGNAQYRFL*HGSGSG*TNSRGCLLAGLSTETGGHYICFNKTA*ENFSRNL*R*N*VSTGESVFYYQSQV*GWTQYSY

                 310       320       330
ndhK         KSLVS-------------SHELLK*-------------
ndhK-pseudo  WKL*SRITLSIAIYVRDLS*NIFKIQKFSIFTRIAEVX
```

```
                  10        20        30        40        50        60        70        80        90       100
rbcL         MSPQTETKASVGFKAGVKDYKLTYYTPEYETKDTDILAAFRVTPQPGVPPEEAGAAVAAESSTGTWTTVWTDGLTSLDRYKGRCYHIEPVAGEENQYIAY
rbcL-pseudo  MSPQTETKASVGFKAGVKDYKLTYYTPEYETKDTDILAAFRVTPQPGVPPEEAGAAVAAESSTGTWTTVWTDGLTSLDRYKGRCYHIEPVAGEENQYIAY

                 110       120       130       140       150       160       170       180       190       200
rbcL         VAYPLDLFEEGSVTNMFTSIVGNVFGFKALRALRLEDLRIPVAYVKTFQGPPHGIQVERDKLNKYGRPLLGCTIKPKLGLSAKNYGRAVYECLRGGLDFT
rbcL-pseudo  VAYPLDLFEEGSVTNMFTSIVGNVFGFKALRALRLEDLRIPVAYVKTFQGPPHGIQVERDKLNKYGRPLLGCTIKPKLGLSAKNYGRAVYECLRGGLYFT

                 210       220       230       240       250       260       270       280       290       300
rbcL         KDDENVNSQPFMRWRDRFLFCAEAIYKSQAETGEIKGHYLNATAGTCEEMMKRAVFARELGAPIVMHDYLTGGFTANTSLAHYCRDNGLLLHIHRAMHAV
rbcL-pseudo  KDDENVNSQPFMRWGDRFLFCAEAIYKAQAETGEIKGHYLNATAGTCEDMMKRAVFARELGVPIVMHDYLTGGFTANTSLAHYCRDNGLLLHIHRAMHAV

                 310       320       330       340       350       360       370       380       390       400
rbcL         IDRQKNHGIHFRVLAKALRMSGGDHIHSGTVVGKLEGERDITLGFVDLLRDDFIEKDRSRGIYFTQDWVSLPGVLPVASGGIHVWHMPALTEIFGDDSVL
rbcL-pseudo  IDRQKNHGIHFRVLAKALRMSGGDHIHSGTVVGKLEGERDITLGFVDLLRDDFIEKDRSRGIYFTQDWVSLPGVLPVASGGIHVWHMPALTEIFGDDSVL

                 410       420       430       440       450       460       470       480
rbcL         QFGGGTLGHPWGNAPGAVANRVALEACVQARNEGRDLAREGNEIIREACKWSPELAAACEVWKEIKFEFEAMDTL*-------------
rbcL-pseudo  QFGGGTLGHPWGNAPGAVANRVALEACVQARNEGRDLAREGNEILREACKWSPELAAACEVWKEIQFDF-I*DSHFFRIVLRVRTRKR*
```
