# Supplementary material for: The Mitogenome of Sedum plumbizincicola (Crassulaceae): Insights into RNA Editing, Lateral Gene Transfer, and Phylogenetic Implications
Source: Biology (Basel). 2022 Nov 13;11(11):1661. doi: 10.3390/biology11111661 (PMC9687357; doi:10.3390/biology11111661)
Supplement: Supplementary file 1 [file biology-11-01661-s001.zip › Figure S5.pdf]

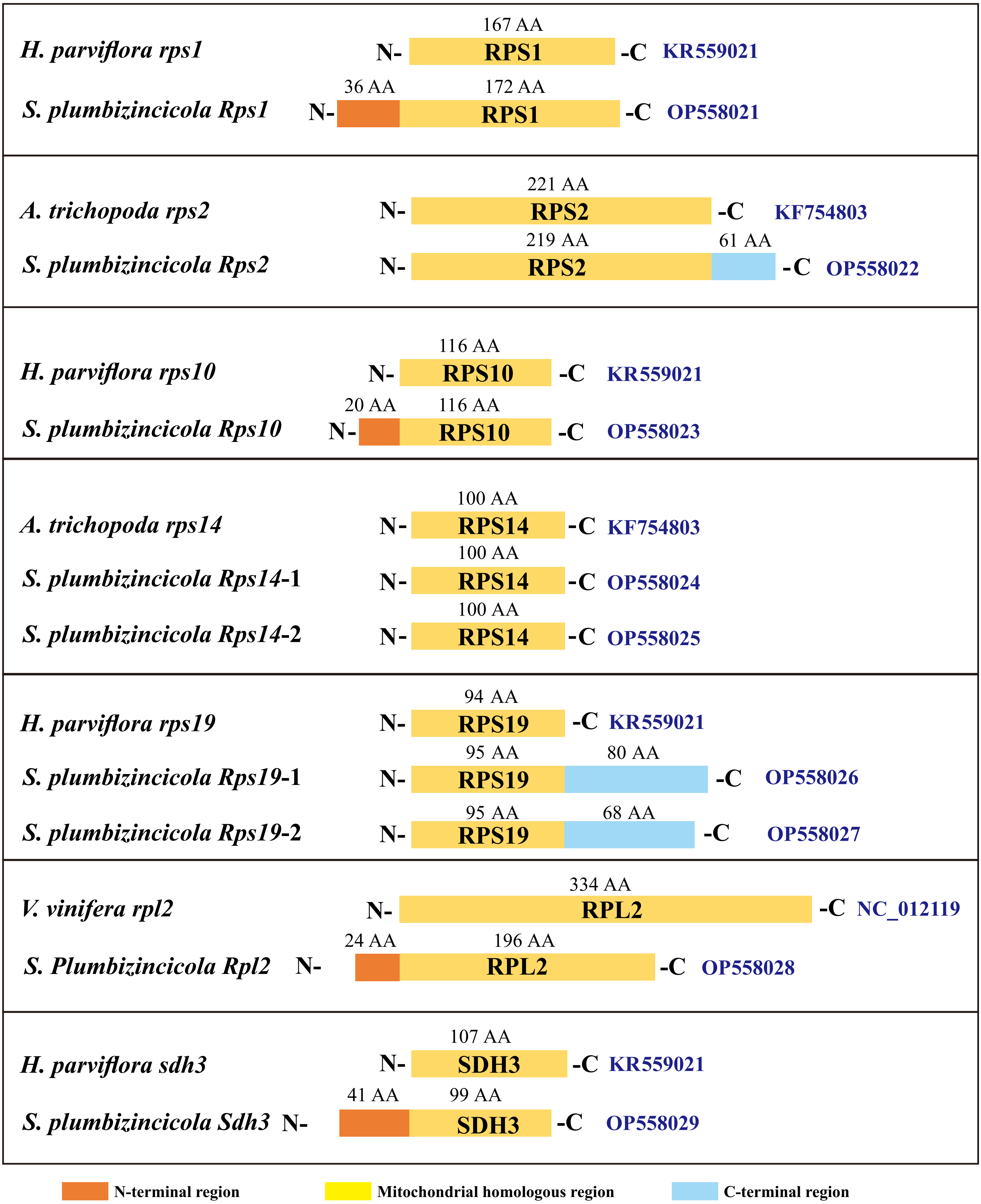
H. parviflora rps1
167 AA
N-
RPS1
-C
KR559021
S. plumbizincicola Rps1
36 AA
172 AA
N-
RPS1
-C
OP558021
A. trichopoda rps2
221 AA
N-
RPS2
-C
KF754803
S. plumbizincicola Rps2
219 AA
61 AA
N-
RPS2
-C
OP558022
H. parviflora rps10
116 AA
N-
RPS10
-C
KR559021
S. plumbizincicola Rps10
20 AA
116 AA
N-
RPS10
-C
OP558023
A. trichopoda rps14
100 AA
N-
RPS14
-C
KF754803
S. plumbizincicola Rps14-1
100 AA
N-
RPS14
-C
OP558024
S. plumbizincicola Rps14-2
100 AA
N-
RPS14
-C
OP558025
H. parviflora rps19
94 AA
N-
RPS19
-C
KR559021
S. plumbizincicola Rps19-1
95 AA
80 AA
N-
RPS19
-C
OP558026
S. plumbizincicola Rps19-2
95 AA
68 AA
N-
RPS19
-C
OP558027
V. vinifera rpl2
334 AA
N-
RPL2
-C
NC_012119
S. Plumbizincicola Rpl2
24 AA
196 AA
N-
RPL2
-C
OP558028
H. parviflora sdh3
107 AA
N-
SDH3
-C
KR559021
S. plumbizincicola Sdh3
41 AA
99 AA
N-
SDH3
-C
OP558029
N-terminal region
Mitochondrial homologous region
C-terminal region
