# Supplementary figures and images for: The Mitogenome of Sedum plumbizincicola (Crassulaceae): Insights into RNA Editing, Lateral Gene Transfer, and Phylogenetic Implications
Source: Biology (Basel). 2022 Nov 13;11(11):1661. doi: 10.3390/biology11111661 (PMC9687357; doi:10.3390/biology11111661)

Recombination via LDRs

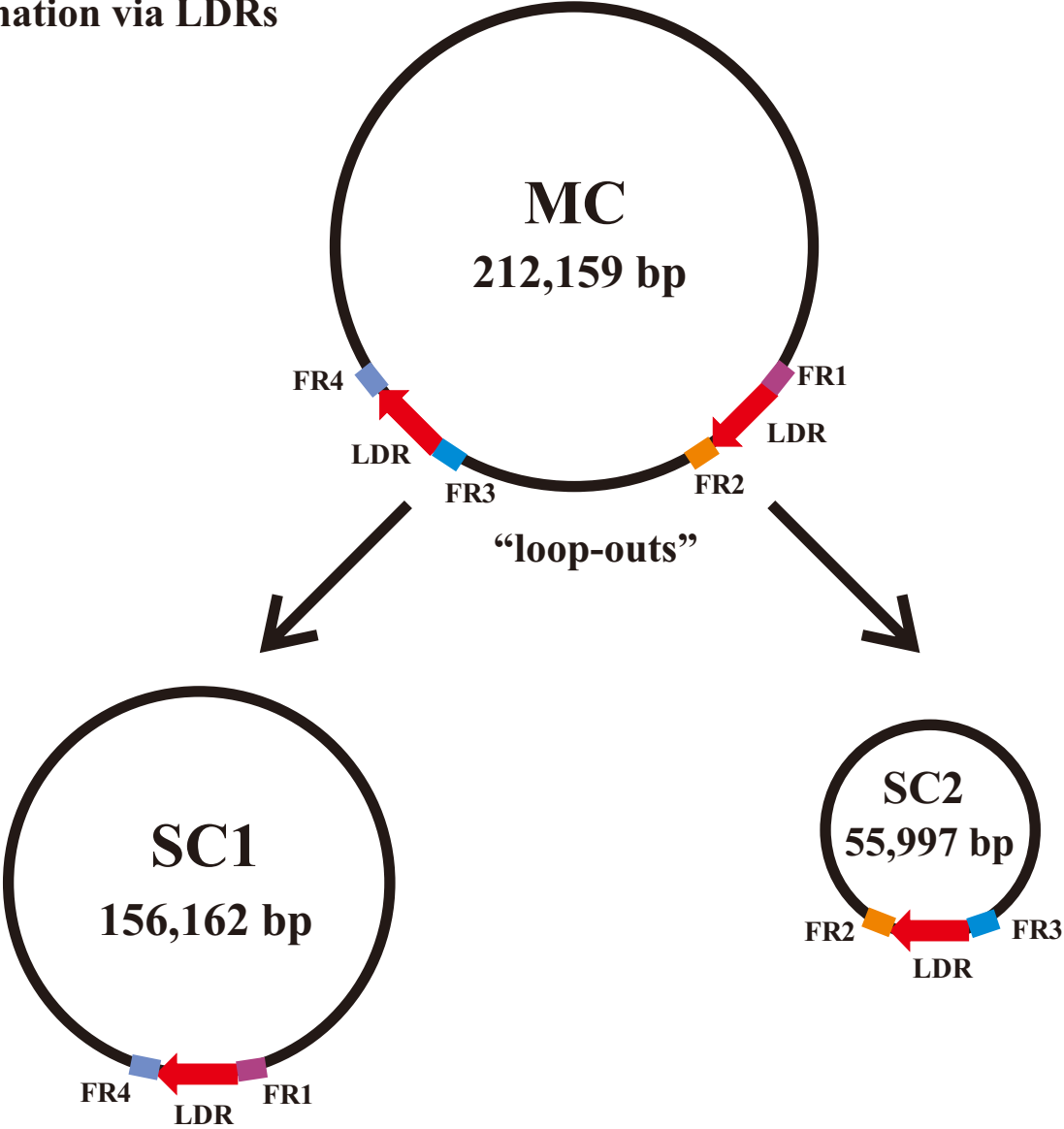

Four types of boundary sequences

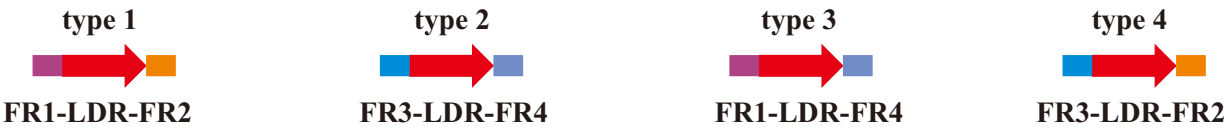

Supplement: Supplementary file 1 [file biology-11-01661-s001.zip › Figure S2.pdf]

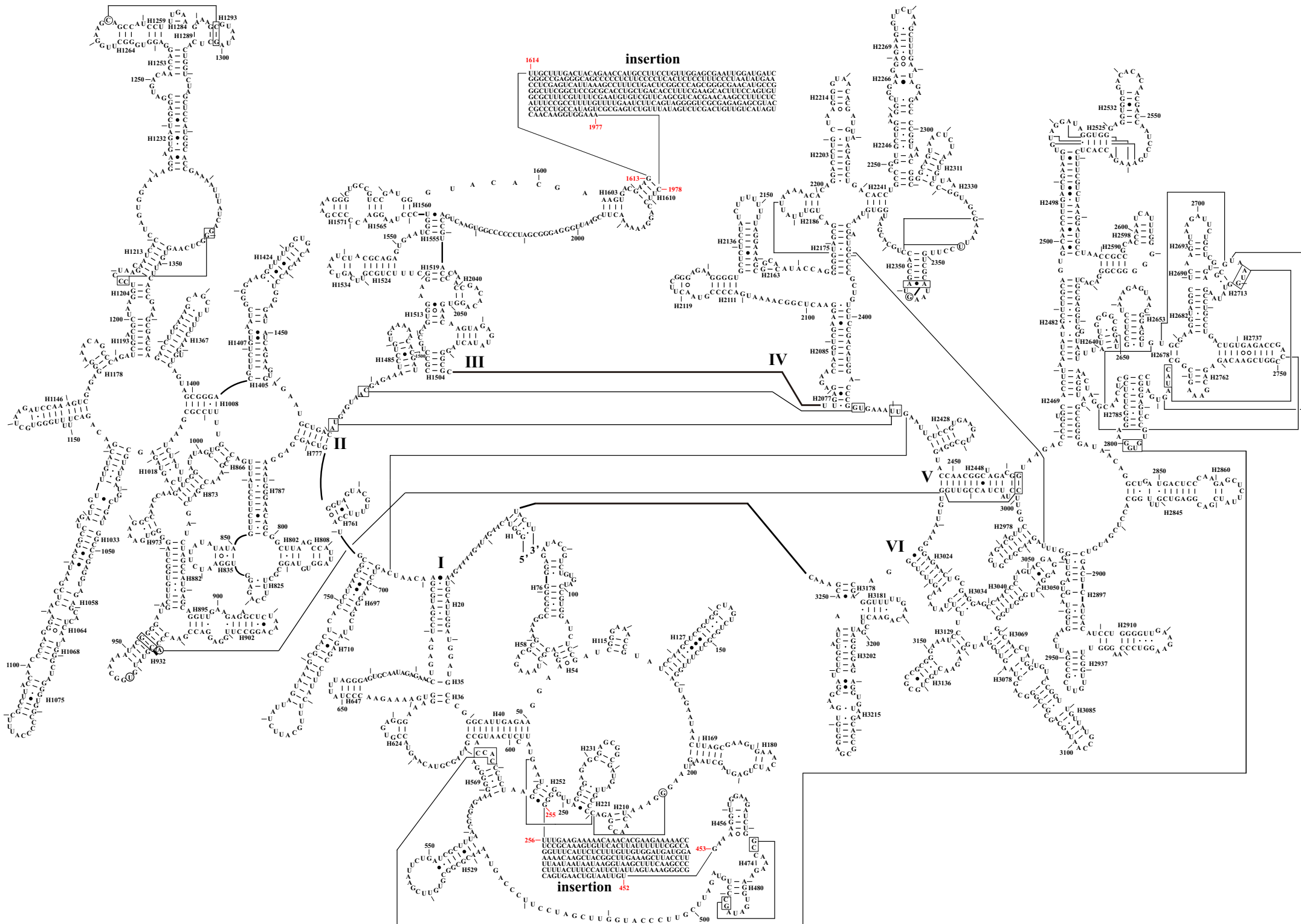

Supplement: Supplementary file 1 [file biology-11-01661-s001.zip › Figure S6.pdf]
