# Supplementary material for: The Mitogenome of Sedum plumbizincicola (Crassulaceae): Insights into RNA Editing, Lateral Gene Transfer, and Phylogenetic Implications
Source: Biology (Basel). 2022 Nov 13;11(11):1661. doi: 10.3390/biology11111661 (PMC9687357; doi:10.3390/biology11111661)
Supplement: Supplementary file 1 [file Figure S4.pdf › Figure S4.pdf]

```
                     10        20        30        40        50        60        70        80        90       100
             ....|....|....|....|....|....|....|....|....|....|....|....|....|....|....|....|....|....|....|....|
matR         MRDLINYCKRKGLLIELGGEAILVIRSERGLAPLKTHYLIRICYARYADDSLLGIVGAVELIIEIQ--------------K--RIAH--FLQSGLNLWVG
matR-pseudo  MRDLINYCKRKGLLIELGGEAILVIRSERGLAPLKTHYLIRICYARYADDSLLGIVGAVELIIEIQNVSPTSYNPPEPLGRLCRINNKSFTE*GRIPRYG

                    110       120       130       140       150       160       170       180       190       200
             ....|....|....|....|....|....|....|....|....|....|....|....|....|....|....|....|....|....|....|
matR         SAGSTTRASRSKVEFLGTVIREVPPRTTPIQFLRELEKRLRVKHRI-HITACHL-RSAIHSKFRNLGNSIPIKQLTKGMSETGSLLDAVQLAETLGTAGV
matR-pseudo  HSGSPSE-DDSHTI-LARA-GEASTGKAPYPY-NCLPPTLRHPFQV*EPR**YPDQTADEGDERN-GESTG-RGSTSGDS-WNSWSQKSQSERIMGRRQA

                    210       220       230       240       250       260       270       280       290       300
             ....|....|....|....|....|....|....|....|....|....|....|....|....|....|....|....|....|....|....|
matR         KSPKVSVLWGAVKHIRQGSRGISLLHSSGRGNVTSDVQEAVSRSGMSVRKLSLYTPAGRKAAGEGGGHWA-GSISNE-FPIEIEAPIKKILRRLRDRGLI
matR-pseudo  HPARIKRDL-VVA*LR*GQRDI----GRSRGSLT---------IGHECPEVVIVY-S-RGSEGGGGRRGTLGRIYRQRIPIEIEAPIKR-YSKASDRGLI

                    310       320       330       340       350       360       370       380       390       400
             ....|....|....|....|....|....|....|....|....|....|....|....|....|....|....|....|....|....|....|
matR         SRRRPWPIHVACLTNVSDGDIANWSAGIAISPLSYYRCRDNLYQVRTIVDYQIRWSAIFTLAH-KKKSS-ARNIIPKYSKDSNIVKKEGGKTLAEFPNSI
matR-pseudo  S--Q-------------E-DLADPRGSLI*RQ*RRHRKLVRGHRDKSSVLLQMPRQPLPSPNYCRLPDPLVCNIHPS-AQEKILVAEYNPKVLQRL*YSE

                    410       420       430
             ....|....|....|....|....|....|..
matR         ELGKLGPGQDPNNKEY*---------------
matR-pseudo  KRRW*DPCRVPQQHRAWQARTRSRSEQQGVLX
```

```
                      10        20        30        40        50        60        70        80        90       100
              ....|....|....|....|....|....|....|....|....|....|....|....|....|....|....|....|....|....|....|....|
ccmFC         MVQLHNFFFFITSMVVPRGTAAPVLLKW--FVSRDVPTGAPSSNGTLIP-IP-IPS-FPLLVY-LHS---R-KF--IRSMDG-AKSGVLLRASRP---T-
ccmFC-pseudo  ......-...-------------S..P.SC..AQQH.YY*NG.SVEMF.RV.LL.MVL*F.FLS...LFWSIYIPE.HTLH.RS.KWS.AQSKP.YRHNW

                     110       120       130       140       150       160       170       180       190       200
              ....|....|....|....|....|....|....|....|....|....|....|....|....|....|....|....|....|....|....|
ccmFC         DIIGRSSSETRARNASFRFVPLLHFIILESKRDFSYLESLCGVLRLLFFRTLFSLPRDRSAKREQARRRKGQILRQNGNEQRRNDKMRCPG-HPHLERRV
ccmFC-pseudo  EKLI.N*.*K.L--I...SPSAF.YS*IQEGLLIFRIFMR.AP.TI.SDSI..T-T.-*VV....VW........K--T.*ATI*MT.*GAR..I*KEGL

                     210       220       230       240       250       260       270       280       290       300
              ....|....|....|....|....|....|....|....|....|....|....|....|....|....|....|....|....|....|....|
ccmFC         EGFWSVAFPVPPSSGGACGATGGKVPPEIGLEALALPTSRQLMAVGHDYYQKAPMKMNISHGGVCICMLGVLLSNTKKIQFTQRLPLGSELHMGKERCCL
ccmFC-pseudo  KV.GL*LS.S.LRRVVLV.PPEARCHLKS..KLSPYQRADS*WLLVTTTTK.LQ*R-*.F.MEE.ASVCW.FFVSAT-AAY-V.-.VAHA.FV--R.AA*

                     310       320       330       340       350       360       370       380       390       400
              ....|....|....|....|....|....|....|....|....|....|....|....|....|....|....|....|....|....|....|
ccmFC         RGIDHLHGPTSHSICGNFLIYKPSLTNDRLMFEHDESLRTDLLPKSFPALYENGKQEHFLHRWMQNREHNNFCLTMFPEKRYFRETTSTTEVAIHTNSFT
ccmFC-pseudo  TRL.PVFN.LLR--.-S--.DSLT.IE.G-*.-NNSCI.---V-SN.G*TNA..LRASL.GN-T.C*P.*E------TR..R*.QLAKWR*ASLAVRK*R

                     410       420       430       440       450       460       470       480       490       500
              ....|....|....|....|....|....|....|....|....|....|....|....|....|....|....|....|....|....|....|
ccmFC         DLYASIGTSSSRTGGWYTTIMKLPFLFFIRIGFMLASSG--GSRSLLRQL-QKD-KLGF*-----------------------------------------
ccmFC-pseudo  S**YHL--RPY.S-S.SRSRIQ-.N.*PGNG.IPTTAG.PA.P*ACGNG.PK.PAQ..QHRMRGWP*CCVGKANFGSWWGKADVGTRIGMQQNS*SHSI*
```
